# Coat characteristics, physiological traits, serum metabolites, and thyroid hormones of Canindé and Moxotó goats in a semiarid environment

**DOI:** 10.1007/s11250-026-05173-9

**Published:** 2026-06-26

**Authors:** Wallace S. Tavares da Silva, Robson M. Freitas Silveira, Jacinara H. Gurgel Morais Leite, Concepta McManus, Débora A. Evangelista Façanha

**Affiliations:** 1 Department of Animal Science, Federal Rural University of the Semi-Arid (UFERSA), Mossoró, RN Brazil; 2 Department of Biosystems Engineering, “Luiz de Queiroz” College of Agriculture, University of São Paulo (ESALQ/USP), Piracicaba, SP Brazil; 3 Department of Animal Science, Federal University of Paraíba (UFPB), Areia, PB Brazil; 4 Center for Nuclear Energy in Agriculture (CENA), University of São Paulo (USP), Piracicaba, SP Brazil; 5 University of International Integration of Afro-Brazilian Lusophony (UNILAB), Redenção, CE Brazil

**Keywords:** Animal adaptation, Canindé goats, Moxotó goats, Thyroid hormones, Coat traits

## Abstract

This study evaluated the adaptive responses of dark-coated Canindé and light-coated Moxotó goats reared under semiarid conditions in Brazilian semiarid through an integrated assessment of thermoregulatory, biochemical, hormonal, and morphological variables. Sixty non-pregnant and non-lactating goats (30 Canindé and 30 Moxotó) were evaluated during summer and winter under the same grazing and management conditions. Animals received native pasture and concentrate supplementation (200 g/head/day). Significant breed × season interactions (*P* < 0.05) were observed for physiological, metabolic, hormonal, and coat variables. Dark-coated Canindé goats showed higher coat surface temperature and greater seasonal variation in coat thickness (0.60 vs. 0.51 mm), as well as marked seasonal changes in T4 (1.67 vs. 4.47 µg/dL), glucose (48.87 vs. 105.03 mg/dL), and albumin (4.42 vs. 8.19 mg/dL) from summer to winter, indicating greater metabolic flexibility. In contrast, light-coated Moxotó goats maintained lower and more stable thyroid hormone concentrations across seasons (T4: 1.63 vs. 1.63 µg/dL) and exhibited increased creatinine levels during winter (0.95 vs. 1.57 mg/dL), suggesting a more conservative adaptive strategy. Multivariate analyses demonstrated distinct adaptive profiles between breeds. Factor analysis revealed integration between thermoregulation and metabolism in Canindé goats, whereas Moxotó goats showed stronger biochemical compensation mechanisms. Decision tree analysis identified globulin and coat thickness as the main discriminating variables between breeds and seasons. These findings demonstrate that locally adapted goat breeds use distinct but effective adaptive strategies to cope with semiarid environmental conditions and provide important phenotypic indicators to support conservation programs, breed selection, and climate-resilient small ruminant production systems.

## Introduction

Adaptation of farm animals to harsh environmental conditions is a crucial factor for determining the viability of livestock systems in tropical and semiarid regions. In environments with high ambient temperatures, intense solar radiation, and prolonged water scarcity, maintaining homeostasis requires complex adjustments involving thermoregulatory mechanisms, physiological responses, blood metabolites related to energy metabolism, thyroid hormone regulation, and morphological coat characteristics that facilitate heat dissipation (Sejian et al. 2018; Silveira et al. 2026). In this context, local breeds subjected to natural selection for centuries, such as native goats from Northeastern Brazil, represent strategic genetic resources to enhance productivity and resilience under heat stress, due to their phenotypic plasticity (Mascarenhas et al. 2022; Silva et al. 2025).

In semiarid regions, small ruminant farming plays a strategic socioeconomic role, but it is constrained by extreme climatic conditions that compromise the performance of specialized breeds (Ferreira et al. 2021). The maintenance of homeothermy in these environments depends on interactions among coat characteristics, physiological responses, endocrine adjustments, and biochemical mechanisms associated with energy balance and heat defense (Camerro et al. 2016; Carabaño et al. 2019). Although rectal temperature and respiratory rate are useful indicators of heat-stress tolerance, they do not fully capture the complexity of adaptive processes. Coat color is a key factor: dark-coated animals absorb more radiation, requiring greater activation of heat-dissipating mechanisms, while light-coated animals reflect part of the solar radiation, leading to different adaptive strategies. Façanha et al. (2022) demonstrated that indigenous ewes with different coat colors (white, red, and black) exhibit significant variations in thermoregulatory and acid–base responses but can recover physiological equilibrium after heat stress. Similarly, Ferreira et al. (2021) reported that Brazilian black goats and ewes raised in an equatorial semiarid environment exhibit distinct thermoregulatory and electrolyte adjustments, highlighting that coat color affects adaptive responses, even in locally adapted breeds.

The adaptability of goats to hot environments cannot be explained by individual variables but rather results from the interaction of physiological, metabolic, hormonal, and morphological heat-defense mechanisms (Vasconcelos et al. 2020; Ribeiro et al. 2015). In this regard, multivariate approaches have proven crucial for characterizing adaptive profiles. Ribeiro et al. (2015 demonstrated that morphological and physiological variables alone were insufficient to distinguish adaptive profiles, whereas integrating physiological, biochemical, hematological, and hormonal variables identified biomarkers with high discriminatory power. These findings show that adaptation is a multifactorial phenomenon and that combining variables offers greater explanatory power than isolated indicators.

Despite advances with other species and local breeds, such as studies with Sindi cattle (Vasconcelos et al. 2020) and Morada Nova sheep few investigations have adopted an integrative approach to evaluate native goat breeds in the Brazilian semiarid region. The Canindé and Moxotó goats are important native genetic resources from Northeastern Brazil that have undergone natural selection for centuries under harsh semiarid conditions, resulting in high rusticity and adaptive capacity to environments characterized by high temperatures, intense solar radiation, and seasonal water scarcity. Canindé goats are typically characterized by a predominantly black coat with contrasting white, cream, or brown markings, dark skin pigmentation, short hair, and well-developed body structure, whereas Moxotó goats are small- to medium-sized animals with a predominantly white coat, a characteristic black dorsal stripe extending from the head to the backline, dark extremities, short smooth hair, and dark skin and mucosa (Ribeiro et al. 2004). Both breeds also present morphological traits associated with adaptation to hot climates, including short hair coat, resistant hooves, and body conformations that favor heat dissipation and survival under semiarid conditions (da Silva et al. 2026). These phenotypic and morphological characteristics may contribute to distinct thermoregulatory, metabolic, and physiological adaptive strategies in response to heat stress.

We hypothesized that, although both breeds are hardy and well-adapted to thermal stress, coat color significantly influences their adaptive responses: Canindé goats (dark coat) would rely more heavily on hormonal and metabolic mechanisms to sustain homeostasis, whereas Moxotó goats (light coat) would show greater physiological stability due to their higher capacity to reflect solar radiation, revealing contrasting but equally effective adaptive strategies to semiarid conditions.

Therefore, this study aimed to characterize the adaptive profiles of Canindé and Moxotó goats to summer and winter climatic conditions by jointly evaluating thermoregulatory responses, biochemical variables, serum thyroid hormones, and coat traits.

## Materials and methods

### Study site and period

The study was conducted on a commercial farm in Lajes (microregion of Angicos), Rio Grande do Norte, Brazil (5°42′00″ S; 36°14′41″ W; altitude: 916 m a.s.l.). The region has two seasons: winter (January–May) and summer (July–September). In the study year, total rainfall was 550 mm, ranging from 400 to 700, with most precipitation concentrated in February and March, and no rainfall was recorded in October. According to the Köppen classification, the area is semi-arid (Alvares et al. 2013). All animals were raised under the same grazing and environmental conditions throughout the experimental period, ensuring that physiological differences reflected breed-specific adaptive responses rather than management-related variation.

### Animals

**Fig. 1:**
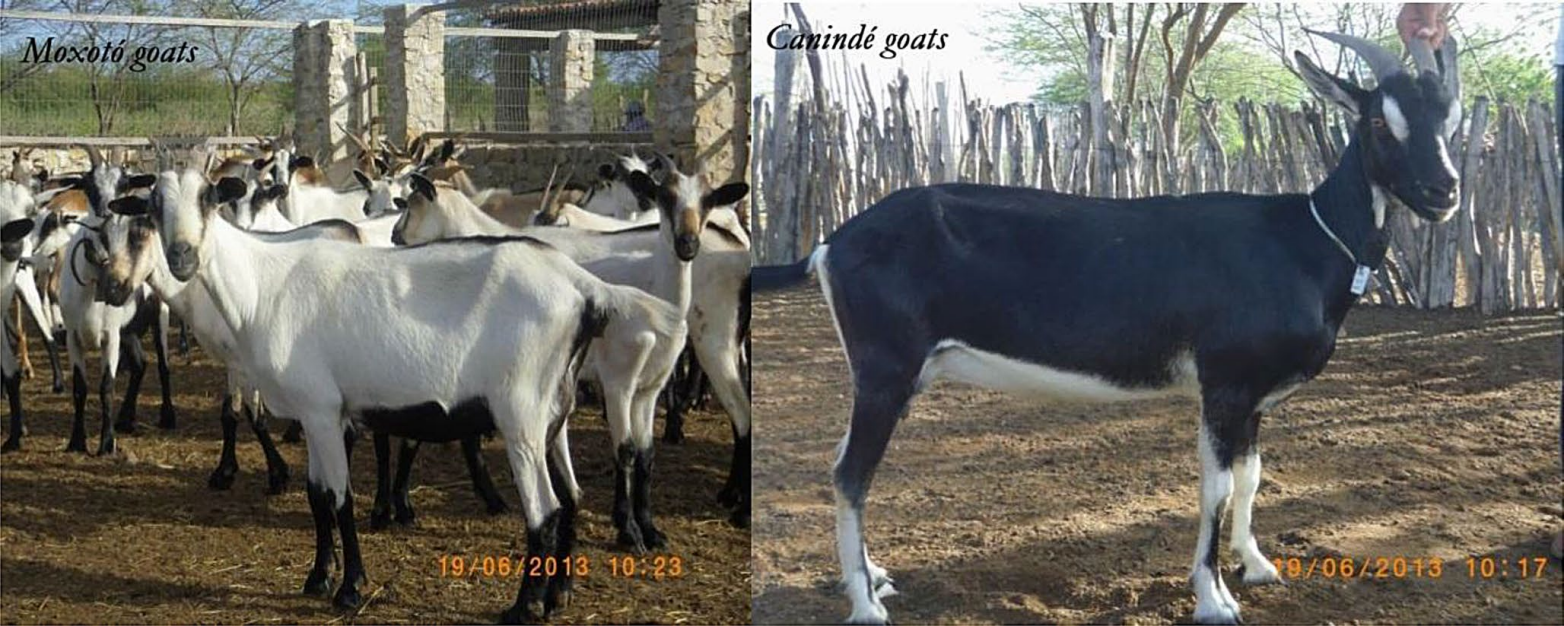
On the left, Moxotó goats, characterized by a predominantly white coat, a black dorsal stripe, and dark extremities. On the right, a Canindé goat, with a predominantly black coat and contrasting white markings

Data were collected from 60 non-lactating, non-pregnant, healthy goats: 30 Canindé and 30 Moxotó (Fig. 1). All goats met the phenotypic and morphological standards of the Associação Norte-Riograndense de Criadores de Caprinos e Ovinos (ANCOC) for their respective breeds. Body condition score ranged from 3 to 4 (on a 1–5 scale). Mean body weight was 35.78 ± 1.74 kg for Canindé and 34.92 ± 2.34 kg for Moxotó. Pregnancy status was confirmed by ultrasonography, and the same individually identified goats were sampled in March and September. All goats received routine sanitary management, including vaccination and deworming, according to local veterinary recommendations.

Goats were managed under the same extensive grazing system on native hyperxerophilous Caatinga pasture throughout the experimental period, with grazing lasting approximately 9 h/day and a low stocking rate typical of semiarid production systems in northeastern Brazil. The grazing area was predominantly composed of native shrubs, grasses, and drought-tolerant herbaceous species typical of the Brazilian semiarid region. Animals were managed daily by an experienced goat keeper. All goats were dewormed and individually identified at the beginning of the experiment, and the same individuals were sampled in March and September. In addition to grazing, goats received a commercial concentrate based on ground corn and soybean meal with a vitamin–mineral premix (≈ 19% crude protein), supplied once daily at 200 g/head/day for maintenance only during both seasons. Mineral salt and water were provided ad libitum throughout the experimental period.

### Data collection

Data were collected in March (winter) and September (summer). During each sampling, measurements followed a consistent order fixed sequence: (i) physiological variables (rectal temperature, respiration rate, and coat surface temperature) were recorded first; (ii) blood sampling was then performed; and (iii) morphological/coat assessments were taken last. Climatic data were recorded concurrently with each sampling. Samples were collected at approximately 09:00 h, before the animals were released for grazing, following the management routine adopted on the farm.

### Environmental variables

Ambient temperature and relative humidity were measured using a digital thermohygrometer and a black-globe thermometer, both positioned at the height of the animals in the same environment where they were maintained, including the grazing and resting areas. From these readings, the Black Globe Humidity Index (BGHI) and Radiant Heat Load (RHL) were calculated according to Silva (2008; Table 1):

1 $$\:BGHI\:=\:\left[\right(TGN\:-\:273.15)\hspace{0.17em}+\hspace{0.17em}0.36\:\times\:\:Tdp\hspace{0.17em}+\hspace{0.17em}41.5]$$

where: TGN = black globe temperature (K); Tdp = dew point temperature (°C); 41.5 = constant.

2 $$\:RHL\hspace{0.17em}=\hspace{0.17em}1.053\:\times\:\:hc\:\times\:\:(TGN\:-\:Ta)\:+\:TGN^4\:(W/m^2)$$

where: hc = convective heat transfer coefficient (W/m²·K); TGN = black globe temperature (K); Ta = air temperature (K); σ = Stefan–Boltzmann constant (5.6697 × 10⁻⁸ W/m²·K⁴).

**Table 1:** Environmental conditions during summer and winter experimental periods

Variables	Summer		Winter	
RHL	624.08	9.16	608.78	7.80
BGHI	89.75	0.65	88.15	0.49

RHL = radiant heat load (W m⁻²); BGHI = black globe humidity index. Values are presented as mean ± standard error

Wind speed was measured using a digital instantaneous anemometer (resolution: 0.01 m/s), connected to a datalogger with a storage capacity of up to 2000 records. Black globe thermometers were constructed of copper (15 cm in diameter, 5 mm in wall thickness), painted with a high-absorbance black coating, and fitted with a central digital thermometer.

### Thermoregulatory responses

Rectal temperature (RT) was measured using a digital clinical thermometer (range: up to 44 °C), inserted 5 cm into the rectum for 2 min. Coat surface temperature (CST) was obtained using a handheld infrared thermometer, aimed at the flank region from a fixed distance of 15 cm, with the sensor held perpendicular to the coat. Respiratory rate (RR) was measured with a stethoscope by counting thoracic movements for one minute, with minimal animal handling.

### Biochemical and hormonal variables

Blood samples (~ 4 mL per goat) were collected from the jugular vein into vacuum tubes containing 0.05 mL of 10% disodium EDTA. Samples were stored on ice immediately after collection and transported to the Laboratory of Experimental Anesthesiology at the Federal Rural University of the Semi-Arid for analysis.

Plasma was separated by centrifugation (Centribio centrifuge, 200 rpm, 10 min), and the supernatant was stored at − 20 °C. Biochemical variables were measured with commercial kits (Vida Biotecnologia, Brazil) and an automated biochemical analyzer (HumaStar 80). Assays included: glucose (enzymatic–colorimetric), cholesterol (enzymatic–colorimetric), triglycerides (enzymatic–colorimetric), urea (GLDL method), creatinine (alkaline picrate method), total protein (Biuret method), albumin (bromocresol green method), AST (kinetic–UV), and ALT (kinetic–UV). Globulin was calculated by subtracting albumin from total protein. Finally, serum thyroid hormones T3 (triiodothyronine) and T4 (thyroxine) were measured using commercial ELISA kits (AccuBin) in an automated analyzer (Elisys Uno).

### Morphological traits of the coat

Hair samples were collected from the dorsal region below the vertebral column using a flat-nose plier and stored in labeled plastic envelopes. Coat thickness was measured in situ using a metal ruler inserted perpendicularly to the skin until reaching the epidermis.

Hair density was estimated by counting the number of hairs in the sampled area (0.207792 cm², corresponding to the plier’s opening area) and extrapolating to hairs/cm² of skin. Average hair length was determined by measuring the 10 longest hairs from each sample with a digital caliper (Udo 1978). Mean hair diameter was measured using a digital micrometer according to the procedure described by Lee (1953).

### Statistical methods

The statistical analyses were conducted using IBM SPSS Statistics v29 (IBM Corp., Armonk, NY, USA), with a significance level set at 5%. Initially, assumptions of residual normality (Shapiro–Wilk test) and homogeneity of variances (Levene’s test) were verified. The presence of outliers was assessed through z-scores and Mahalanobis distance in multivariate models.

Sample size adequacy was verified using power analysis in the SPSS GLM Power module, considering breed, season, and their interaction as fixed effects, with α = 0.05 and a minimum statistical power of 80% to detect interaction effects.

For each physiological, biochemical, or morphological variable, data were analyzed using a repeated-measures factorial analysis of variance (ANOVA), considering breed, season, and their interaction as fixed effects, with repeated measurements obtained from the same animals across seasons.

3 $${Y_{ijk}} = \mu + {B_i} + {S_j} + ({B_i} \times \>{S_j}) + {e_{ijk}}$$

Where: Y_ijk_ represents the observed response, µ the overall mean, B_i_ the fixed effect of breed (Canindé or Moxotó), S_j_ the fixed effect of season (summer or winter), B_i_×S_j_ the interaction between breed and season, and e_ijk_ the random error, assumed to be normally distributed. When the interaction was significant, simple effects were investigated, comparing breeds within each season and seasons within each breed. Results are presented as mean ± standard error of the mean (SEM), and significance was determined based on the ANOVA F-test for the effects of breed, season, and their interaction.

To investigate the latent structure and interrelationships among physiological, biochemical, hormonal, and morphological variables, an exploratory factor analysis was performed separately for each breed. This approach reduced data dimensionality and enabled the identification of integrated adaptive mechanisms and coordinated biological responses rather than isolated trait-specific effects. Sampling adequacy was assessed using the KMO index and Bartlett’s test of sphericity. Extraction was carried out using the principal component method, followed by Varimax rotation, retaining factors with eigenvalues greater than 1, and interpreting factor loadings equal to or greater than 0.40.

Differences among the four groups (Summer–Canindé, Summer–Moxotó, Winter–Canindé, and Winter–Moxotó) were further evaluated using canonical discriminant analysis to determine whether the combined multivariate profile could effectively discriminate the breed × season groups and to identify the variables contributing most strongly to group separation. Independent variables were selected using the stepwise procedure based on minimization of Wilks’ λ. The significance of discriminant functions, canonical correlations, group centroids, and classification accuracy was evaluated, with cross-validation using the leave-one-out method.

The CHAID (Chi-squared Automatic Interaction Detection) algorithm was used to construct decision trees aimed at classifying the groups. This complementary and interpretable classification approach was applied to identify the main predictor variables and threshold values responsible for distinguishing adaptive profiles, generating practical biological decision rules. The dependent variable was categorical (four breed × season groups), and predictors included physiological, biochemical, and morphological variables. The splitting criterion was the chi-squared test with Bonferroni adjustment, adopting *p* ≤ 0.05. The tree was validated using SPSS automatic cross-validation, and the decision rules, main nodes, and classification matrix were reported.

## Results

The analysis of the effects of breed (B), season (S), and the B × S interaction revealed distinct physiological and metabolic patterns between Canindé and Moxotó goats across summer and winter (Table 2). When interactions were significant, results are presented within each breed by season and between breeds within each season.

**Table 2:** Adaptive responses of Canindé and Moxotó goats to climatic conditions in summer and winter in a semiarid region of the Brazilian semiarid region. *n* = 60 goats

Variables	Breed (B)	Season (S)		Mean	SEM	*P*-value		
		Summer	Winter			B	S	B×S
CST	Canindé	39.18Aa	34.95Bb	37.07	3.684	0.009	0.006	0.001
	Moxotó	37.24Bb	39.13Aa	38.18				
	Mean	38.21	37.04					
RT	Canindé	39.74Aa	39.17Ab	39.45	0.678	0.563	< 0.001	< 0.001
	Moxotó	39.61Aa	39.4Ab	39.5				
	Mean	39.67	39.28					
RR	Canindé	49Aa	35.93Ab	42.46	18.199	0.08	< 0.001	< 0.001
	Moxotó	46.56Aa	30.8Ab	38.68				
	Mean	47.78	33.37					
CT	Canindé	0.6Aa	0.51Ab	0.56	0.155	< 0.001	< 0.001	< 0.001
	Moxotó	0.47Ba	0.29Bb	0.38				
	Mean	0.54	0.4					
CL	Canindé	30.15Aa	23.37Ab	26.76	6.927	< 0.001	< 0.001	< 0.001
	Moxotó	30.51Aa	17.68Bb	24.09				
	Mean	30.33	20.52					
HDM	Canindé	0.05Ab	0.06Aa	0.06	0.0315	0.123	0.011	0.014
	Moxotó	0.05Aa	0.05Ba	0.05				
	Mean	0.05	0.06					
T_3_	Canindé	5.37Aa	4.71Ab	5.04	1.3387	< 0.001	0.002	0.002
	Moxotó	3.00Ba	3.00Ba	3				
	Mean	4.19	3.86					
T_4_	Canindé	1.67Ab	4.47Aa	3.07	1.310	< 0.001	< 0.001	< 0.001
	Moxotó	1.63Aa	1.63Ba	1.63				
	Mean	1.65	3.05					
GLU	Canindé	48.87Ab	105.03Aa	76.95	36.359	< 0.001	< 0.001	< 0.001
	Moxotó	57.1Aa	30.5Bb	43.8				
	Mean	52.98	67.77					
CHO	Canindé	131.17Ab	168.83Aa	150	42.169	< 0.001	< 0.001	< 0.001
	Moxotó	111.2Ba	119.03ba	115.12				
	Mean	121.18	143.93					
TP	Canindé	30.87	37.83	34.35 A	13.128	< 0.001	0.358	0.452
	Moxotó	33.32	23.47	28.39B				
	Mean	32.09	30.65					
URE	Canindé	18.93	36.9	27.92	13.257	0.441	< 0.001	0.894
	Moxotó	17.8	36.1	26.95				
	Mean	18.37b	36.5a					
CRE	Canindé	0.85Ba	0.63Bb	0.74	0.4244	< 0.001	< 0.001	< 0.001
	Moxotó	0.95Ab	1.57Aa	1.26				
	Mean	0.9	1.1					
TRI	Canindé	6.97Bb	10.16Aa	8.57	1.614	< 0.001	< 0.001	< 0.001
	Moxotó	10.05Aa	9.34Bb	9.7				
	Mean	8.51	9.75					
ALB	Canindé	4.2Bb	9.30Aa	6.75	2.327	< 0.001	< 0.001	< 0.001
	Moxotó	4.42Ab	8.19Ba	6.31				
	Mean	4.31	8.75					
GLO	Canindé	2.77Ba	0.86Ab	1.81	2.09	< 0.001	< 0.001	< 0.001
	Moxotó	5.62Aa	1.15Ab	3.39				
	Mean	4.2	1.01					
AST	Canindé	103.43	153.03	128.23B	37.672	0.036	< 0.001	0.183
	Moxotó	106.03	164.6	135.32A				
	Mean	104.73b	158.82a					
ALT	Canindé	28.7	38.2	33.45B	9.365	0.029	< 0.001	0.094
	Moxotó	29.2	41.93	35.57A				
	Mean	28.95b	40.07a					

Note: CST = coat surface temperature (°C); RT = rectal temperature (°C); RR = respiratory rate (breaths/min); CT = coat thickness (mm); CL = coat length (mm); HDM = hair diameter (µm); T_3_ = triiodothyronine (ng/mL); T_4_ = thyroxine (µg/dL); GLU = serum glucose (mg/dL); CHO = total serum cholesterol (mg/dL); TRI = serum triglycerides (mg/dL); URE = serum urea (mg/dL); CRE = serum creatinine (mg/dL); TP = total proteins (g/dL); ALB = serum albumin (g/dL); GLO = serum globulins (g/dL); AST = aspartate aminotransferase (U/L); ALT = alanine aminotransferase (U/L)

Means followed by different letters, uppercase in rows (breed comparison) and lowercase in columns (season comparison), indicate significant differences between breed, season, or their interaction effects at *P* < 0.05

### Thermoregulatory responses

A significant breed x season interaction was observed for CST (*P* = 0.001). In summer, Canindé showed higher CST than Moxotó (39.18 vs. 37.24 °C), whereas in winter the opposite occurred (Moxotó > Canindé: 39.13 vs. 34.95 °C), evidencing antagonistic seasonal responses between breeds. RT showed no main effect of breed (*P* = 0.563) but was influenced by season and the B×S interaction (*P* < 0.001). RT decreased in winter in both breeds (Canindé: 39.74→39.17 °C; Moxotó: 39.61→39.40 °C), with no breed differences within seasons. RR followed the same trend, with a breed x season interaction (B×S, *P* < 0.001; B, *P* = 0.08), decreasing in winter for Canindé (49.00→35.93 mov/min) and Moxotó (46.56→30.80 mov/min).

### Morphological and structural traits

Coat thickness (CT) was influenced by breed, season, and their interaction (*P* < 0.001). Canindé consistently had higher values than Moxotó in both summer (0.60 vs. 0.47 mm) and winter (0.51 vs. 0.29 mm), with seasonal reductions in both breeds. Coat length (CL) also showed interaction (*P* < 0.001). During summer, both breeds had similar values (~ 30 mm), while in winter Canindé maintained higher CL than Moxotó (23.37 vs. 17.68 mm), with seasonal reductions in both. For hair diameter (HDM), no main breed effect was detected (B, *P* = 0.123), but season and the B×S interaction were significant (S, *P* = 0.011; B×S, *P* = 0.014): Canindé increased from 0.05 to 0.06 mm between summer and winter, whereas Moxotó remained unchanged (~ 0.05 mm), suggesting greater seasonal plasticity in Canindé goats.

### Thyroid hormones

Triiodothyronine (T_3_) exhibited significant interaction and main effects (B×S, *P* = 0.002; B, *P* < 0.001; S, *P* = 0.002). Canindé goats consistently had higher plasma T_3_ concentrations than Moxotó goats (summer: 5.37 vs. 3.00 ng/mL; winter: 4.71 vs. 3.00 ng/mL), with a seasonal reduction in Canindé but no change in Moxotó goats. Thyroxine (T_4_) was also influenced by breed, season, and B x S interaction (*P* < 0.001). Plasma T_4_ markedly increased in Canindé goats in winter (1.67→4.47 µg/dL), while this hormone remained constant in Moxotó goats (1.63 µg/dL), resulting in clear divergence between breeds in winter.

### Energy and lipid biomarkers

Plasma glucose showed a significant breed × S interaction (*P* < 0.001). In Canindé goats, plasma glucose levels more than doubled in winter, whereas this plasma metabolite decreased in Moxotó goats in winter, revealing opposing seasonal metabolic profiles. For plasma cholesterol, there was a B × S interaction, with the main effect on this metabolite (*P* < 0.001), increasing in both breeds but more sharply in Canindé than in Moxotó. Plasma triglyceride concentrations showed a significant breed × season interaction (*P* <0.001). Moxotó goats presented higher triglyceride concentrations during summer, whereas Canindé goats exhibited higher values during winter.

### Nitrogenous compounds and serum proteins

Plasma total protein concentrations were not affected by season (*P* = 0.358) or by the breed × season interaction (*P* = 0.452). However, a significant breed effect was observed (*P* <0.001) with Canindé goats presenting higher plasma total protein concentrations (34.35 g/L) than Moxotó goats (28.39 g/L). Urea (URE) was affected solely by season (*P* < 0.001), with higher levels in winter than in summer. Plasma creatinine concentrations were affected by main effects and there was a B x S interaction (*P* < 0.001): in Canindé, it decreased in winter, whereas in Moxotó it increased, again showing an opposite seasonal response. Plasma albumin concentrations (ALB) were affected by the breed × season interaction (*P* <0.001) as well as by the main effects of breed (*P* <0.001) and season (*P* <0.001). Albumin concentrations increased from summer to winter in both breeds. During summer, Moxotó goats exhibited higher albumin concentrations than Canindé goats (4.42 vs. 4.20 g/dL), whereas during winter, Canindé goats showed higher values than Moxotó goats (9.30 vs. 8.19 g/dL).  Plasma albumin (ALB) followed a similar pattern (*P* < 0.001): breeds were similar in summer, but in winter, Canindé goats had higher plasma levels, with sharper increases. Plasma globulin concentrations (GLO) also showed a B x S interaction (*P* < 0.001), decreasing in winter for both breeds. In summer, Moxotó goats had higher values than Canindé goats, whereas in winter plasma levels of these metabolites converged.

### Liver enzyme

Plasma aspartate aminotransferase (AST) concentrations were affected by breed and season (B, *P* = 0.036; S, *P* < 0.001). Plasma AST concentrations were higher in winter, with slightly higher levels in Moxotó goats. Alanine aminotransferase (ALT) had similar results (B, *P* = 0.029; S, *P* < 0.001), increasing in winter, with slightly higher averages in Moxotó goats.

### Relationships among variables and seasonal adaptive profile discrimination

**Fig. 2:**
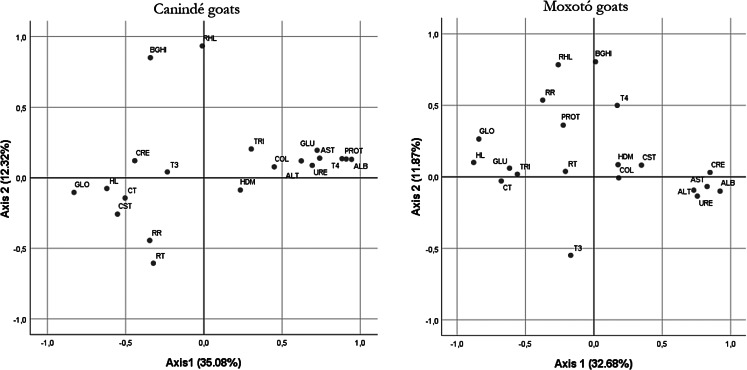
Biplot of the factor analysis of the adaptive profile of Moxotó and Canindé goats in a Brazilian semiarid region. Note: CST = coat surface temperature (°C); RT = rectal temperature (°C); RR = respiratory rate (breaths/min); CT = coat thickness (mm); CL = coat length (mm); HDM = hair diameter (µm); T_3_ = triiodothyronine (ng/mL); T_4_ = thyroxine (µg/dL); GLU = serum glucose (mg/dL); CHO = total serum cholesterol (mg/dL); TG = plasma triglycerides (mg/dL); URE = plasma urea (mg/dL); CRE = plasma creatinine (mg/dL); TP = plasma total proteins (g/dL); ALB = plasma albumin (g/dL); GLO = plasma globulins (g/dL); AST = aspartate aminotransferase (U/L); ALT = alanine aminotransferase (U/L)

Factor analysis showed distinct association patterns among physiological, biochemical, and morphological traits. In Canindé goats, the first two axes accounted for 47.4% of the variance (Axis 1 = 35.1%; Axis 2 = 12.3%; Fig. 2). Energy and protein metabolism variables (glucose, total proteins, albumin, T_4_, and liver enzymes) clustered positively, while thermoregulatory traits (RT, RR, and CST) loaded negatively, suggesting integrated metabolic–thermoregulatory adaptation. Environmental load (BGHI and RHL) contributed to Axis 2, opposing RT and RR, highlighting environmental influence. In Moxotó goats, the first two axes explained 44.6% of the variance (Axis 1 = 32.7%; Axis 2 = 11.9%). Protein–hepatic metabolism (albumin, urea, AST, ALT, and creatinine) loaded positively, while glucose, globulins, triglycerides, and body measurements loaded negatively, indicating dissociation between hepatic/protein and energy metabolism. Axis 2 grouped thermoregulation (RR, RHL, and BGHI) with protein and T_4_, suggesting that increased metabolic/hormonal activity is associated with greater physiological effort under heat stress.

**Fig. 3:**
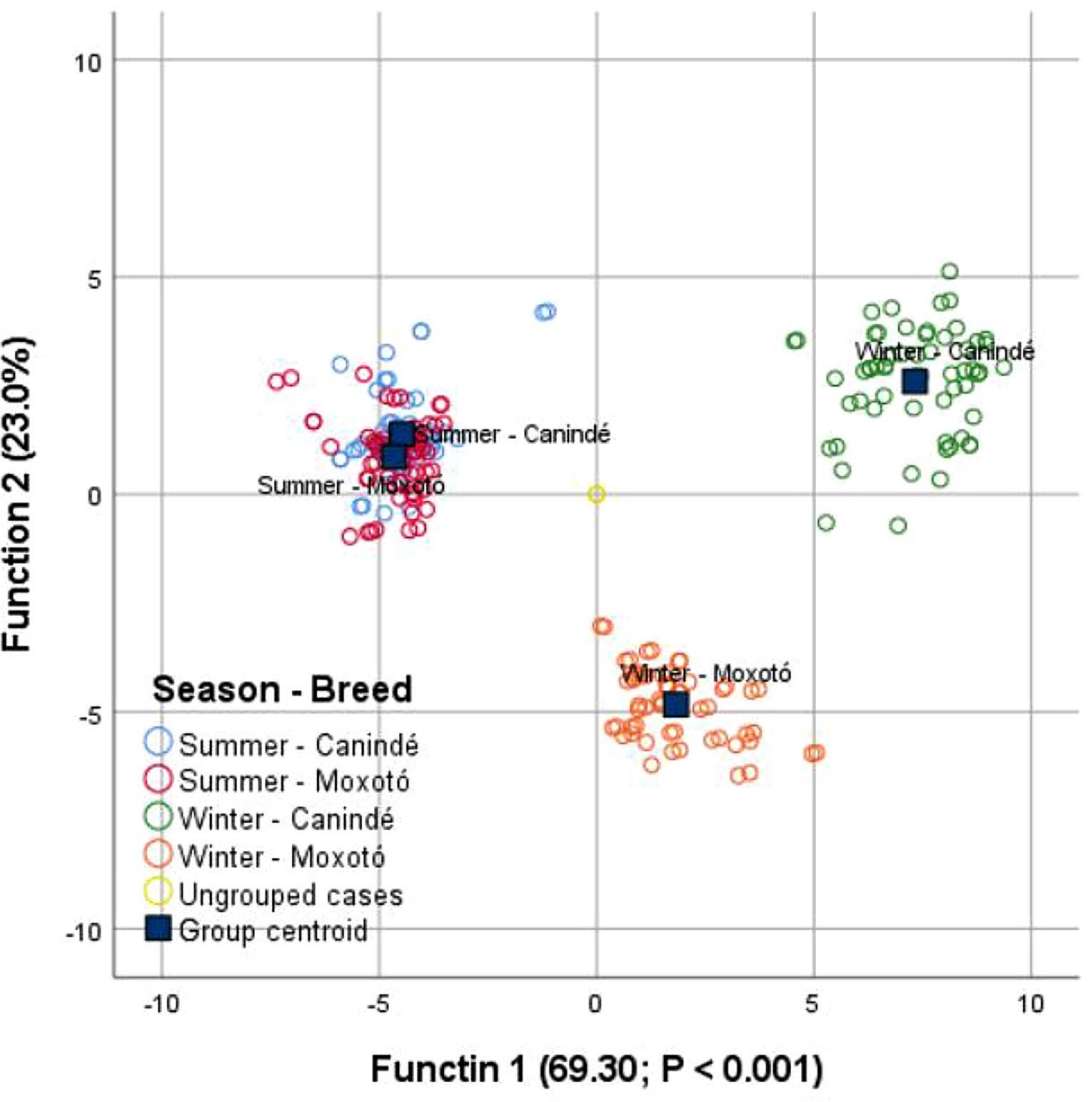
Canonical discriminant analysis (CDA) of the adaptive profile of Canindé and Moxotó goats across seasons (summer and winter) in a Brazilian semiarid region

Canonical discriminant analysis showed differences between breed × season groups (Fig. 3). The first two functions accounted for 92.3% of total variance (Function 1 = 69.3%; Function 2 = 23.0%; *P* < 0.001). Function 1 separated winter from summer, mainly driven by albumin (0.934), T_4_ (0.460), glucose (0.136), and cholesterol (0.165) with positive contributions, and total proteins (–0.239), creatinine (–0.218), cholesterol (–0.165), and RT (–0.215) with negative contributions. This indicates that the winter separation was primarily due to increases in plasma albumin and T4. Function 2 further distinguished Canindé-winter from Moxotó-winter, with positive weights for T_4_ (0.594), creatinine (0.508), and glucose (0.363), and a strong negative contribution of albumin (–0.418).

**Fig. 4:**
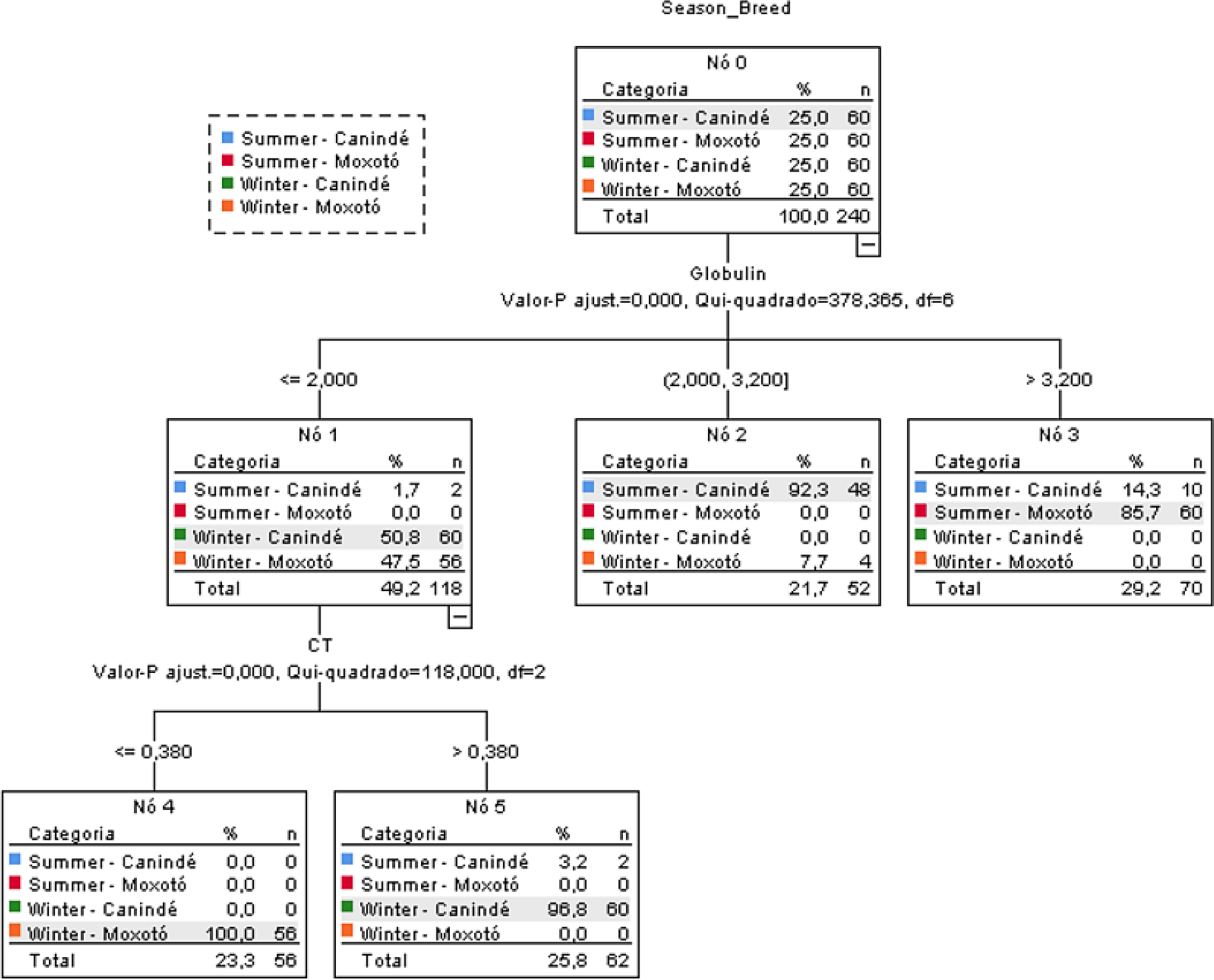
Classification tree analysis of the adaptive profile of Canindé and Moxotó goats in a Brazilian semiarid region, according to season (summer and winter). CT = coat thickness (mm)

Decision tree analysis identified plasma globulin and CT as key discriminants among the four breed × season groups (χ² = 378.4; df = 6; adjusted *P* < 0.001; Fig. 4). The first split was plasma globulin concentration: ≤2.0 g/dL classified mostly Winter–Canindé (50.8%) and Winter–Moxotó (47.5%); 2.0–3.2 g/dL corresponded mainly to Summer–Canindé (92.3%); >3.2 g/dL identified Summer–Moxotó (85.7%). Within the ≤ 2.0 g/dL node, CT further separated winter breeds: CT ≤ 0.380 classified exclusively Winter–Moxotó, whereas CT > 0.380 classified predominantly Winter–Canindé (96.8%).

## Discussion

These findings showed that, although Canindé and Moxotó goats have an adaptive history in the Brazilian semiarid region, the physiological, biochemical, and morphological mechanisms underlying seasonal responses are distinct and complementary. Despite the relatively small annual variation in environmental heat load typical of low-latitude semiarid regions, even subtle seasonal changes were sufficient to modify coat characteristics, thermoregulatory responses, and metabolic activity in both breeds. This differentiation suggests that each breed has developed specific adaptive strategies to cope with high radiant heat loads and periods of nutritional restriction, which may be associated with coat traits such as color, morphology, and hair density.

From a thermoregulatory perspective, rectal temperature remained within the physiological limits described for goats (Ribeiro et al., 2018), regardless of breed or season, confirming the capacity of both groups to maintain homeothermy even under challenging environmental conditions. This pattern has also been reported in studies with native goats in Northeast Brazil (Façanha et al. 2020), highlighting their rusticity. RR, however, increased in summer in both breeds, suggesting activation of the evaporative cooling mechanism (Finch et al. 1980; Silva 2008). Nonetheless, values remained within the range considered as mild heat stress (Silanikove 2000).

Coat surface temperature reflected the combined effects of solar radiation absorption, coat insulation, and heat transfer dynamics. During summer, the predominantly light-colored and thinner coat of Moxotó goats likely reflected a greater proportion of incident solar radiation, limiting surface heating despite the higher environmental heat load. In contrast, the darker coat of Canindé goats absorbed more solar radiation, resulting in higher CST values. During winter, however, the lower coat thickness and reduced insulation capacity of Moxotó goats may have facilitated greater conductive heat transfer from the body core to the coat surface, increasing CST despite the milder environmental conditions. Conversely, the thicker and denser coat of Canindé goats likely acted as a thermal barrier, reducing surface heat dissipation and contributing to lower CST values during winter. These findings indicate that CST is not determined exclusively by ambient temperature, but rather by the interaction between environmental conditions and coat structural properties (Maia et al. 2003; Amorin et al. 2019).

Decision tree analysis reinforced this relationship by identifying plasma globulin and coat thickness as determinants of discrimination between breeds and seasons. Dense long coats act as a barrier to solar radiation but may hinder heat dissipation, whereas shorter, less dense coats favor convection, radiation, and evaporative cooling through sweating and panting (Leite et al. 2020). This explains the greater integration between metabolism and morphology observed in Canindé goats in contrast to the stronger reliance on physiological adjustments in Moxotó goats.

Thyroid hormones played a central role. Canindé goats exhibited consistently higher plasma T_3_ and T_4_ concentrations, with a marked winter increase in T_4_, reflecting greater metabolic mobilization under milder conditions. Animals under heat stress reduce thyroid activity to minimize endogenous heat production (Bianco, 2000; Todini et al. 2007). Therefore, the winter rise in T_4_ in Canindé goats indicates endocrine plasticity, enabling greater metabolic oxidation when heat load is reduced. In contrast, Moxotó goats maintained stable and low plasma T_4_ levels, supporting a conservative strategy of metabolic heat economy, consistent with their rusticity in hot environments.

Energy and lipid biomarkers showed more dynamic profiles in Canindé goats: plasma glucose levels doubled in winter, associated with higher plasma T_4_ and albumin concentrations, indicating greater metabolic activation under milder environmental conditions. Plasma cholesterol also rose more sharply in this breed. Conversely, Moxotó goats showed reduced plasma glucose levels and smaller plasma cholesterol fluctuations in winter, reflecting an energy-saving strategy. Recent studies confirm that heat stress reduces feed intake, lowers serum glucose levels, and increases blood cortisol, NEFA, and urea (Wang et al. 2024). These findings reinforce the view that Canindé goats respond to improved environmental conditions with greater metabolic mobilization, whereas Moxotó goats adopt a parsimonious strategy.

Regarding plasma nitrogenous compounds, urea increased in winter in both breeds, but plasma creatinine exhibited an opposite pattern: it decreased in Canindé goats, suggesting reduced muscle degradation, and increased in Moxotó goats, suggesting greater mobilization of muscle energy reserves to meet energy demands. This reinforces contrasting profiles: Canindé goats rely more on dietary protein, whereas Moxotó depends more on muscle catabolism. Plasma proteins also show seasonality. In summer, Moxotó goats showed higher plasma concentrations of total proteins and globulins, suggesting greater immune activation under heat. In winter, Canindé goats surpassed Moxotó in plasma total protein and albumin concentrations, indicating indicating greater circulating protein concentrations during favorable environmental conditions. The literature indicates that prolonged environmental heat reduces total protein, albumin, and globulin due to decreased feed intake and water redistribution (Wang et al. 2024). Therefore, the increase in Canindé goats during winter reinforces the link between milder environmental conditions and enhanced protein synthesis.

The increase in liver enzymes AST and ALT during winter, especially in Moxotó goats, suggests greater hepatic involvement in seasonal metabolic adjustments. These findings support the idea that Moxotó goats rely on a more fragmented and conservative metabolic strategy compared with Canindé goats. Integrative analyses further reinforce that the adaptive mechanisms of the two breeds reflect distinct physiological and metabolic responses under semiarid conditions. The stronger association between metabolic and thermoregulatory variables observed in Canindé goats suggests a more integrated adaptive response and greater metabolic plasticity to cope with seasonal variations in environmental heat load. In contrast, Moxotó goats appear to depend on more specific metabolic adjustments, particularly related to hepatic and protein metabolism. In this context, the discriminative importance of albumin, T4, globulin, and coat thickness reinforces the central role of metabolic mechanisms and morphostructural traits in the adaptation of native goats to semiarid environments.Although the first two factors explained a moderate proportion of the total variance (44.6–47.4%), these values are consistent with the multifactorial nature of adaptive responses in biological systems, particularly under semiarid conditions where thermoregulatory, metabolic, hormonal, and morphological mechanisms interact simultaneously. In this context, factor analysis should not be interpreted as fully explaining the complexity of adaptation, but rather as an approach capable of identifying the main association patterns and coordinated biological responses underlying seasonal adaptation in each breed. The moderately explained variance also reinforces that adaptive capacity is influenced by multiple interconnected pathways that cannot be entirely summarized by a limited number of latent dimensions, which helps explain why many studies consider climate adaptation a multifactorial process (Sejian et al. 2018; Silveira et al. 2025; 2026).

Decision tree modeling indicated that serum globulin was the primary attribute for discriminating the four groups (summer–Canindé, summer–Moxotó, winter–Canindé, and winter–Moxotó), with values ≤ 2.0 g/dL, almost exclusively clustering winter animals (both breeds) and values > 3.2 g/dL classifying mostly summer–Moxotó. The second branching, based on coat length, separated the breeds in winter. These patterns are consistent with the literature on seasonal rhythms in serum protein fractions in goats, with higher plasma albumin in winter and, consequently, lower globulins during this season, thereby enhancing the discriminant capacity of globulins in cooler conditions (Piccione et al. 2007). Conversely, under summer heat stress, immune-metabolic activation and homeostatic adjustments can elevate or concentrate globulin fractions depending on management and genotype, which helps explain the > 3.2 g/dL branch strongly associated with summer–Moxotó in our dataset.

Recent studies confirm that heat stress consistently alters physiological and biochemical markers (RT, CST, RR, liver enzymes, and serum proteins), but the direction and magnitude of protein changes vary across breeds, environments, and nutritional conditions. For example, in Malabari goats under heat stress, seasonal reductions in blood total protein/ALT and glucose changes were observed, whereas other studies reported seasonal oscillations in serum proteins associated with management and thermal stress levels (Gopikrishnan et al. 2025). In Dazu goats, variations in AST/ALT and energetic variables under different temperature-humidity index levels reinforce the view that the hepatic–metabolic axis is highly sensitive to warm environments and contributes to group separation when integrated into classifiers (Wang et al. 2024).

The second split by coat thickness in the winter node, distinguishing Winter–Moxotó from Winter–Canindé, aligns with evidence that coat/skin traits (thickness, length, density, and thermal properties) are key elements of the adaptive strategies of local goat breeds. Recent studies on indigenous goats demonstrate that tegumentary attributes are targets of thermal selection and correlate with heat responses, supporting the classificatory role of morpho-structural variables in our model (da Silva et al. 2026; Mullakkalparambil Velayudhan et al. 2025).

## Conclusion

This study showed that Canindé and Moxotó goats exhibit contrasting seasonal adaptive profiles in semiarid conditions of northern Brazil, reflecting distinct physiological, metabolic, and morphological strategies for coping with environmental challenges. Canindé goats displayed a more integrated response, in which energy and protein metabolism were coupled with thermoregulatory adjustments, suggesting higher plasticity and metabolic flexibility, particularly during winter when thyroid hormones, plasma glucose, cholesterol, and albumin increased markedly. In contrast, Moxotó goats showed more fragmented responses, prioritizing specific metabolic pathways, such as hepatic protein metabolism, while maintaining relatively stable thyroid activity and less coordinated thermoregulatory adjustments.

The opposing seasonal responses between breeds, especially in key biomarkers such as plasma glucose, creatinine, globulins, and thyroid hormones, emphasize their divergent adaptive strategies. Discriminant and classification analyses confirmed that plasma albumin, T4, globulins, and coat traits were the most revealing variables for distinguishing seasonal adaptive profiles.

Overall, these findings emphasize that Canindé goats rely on a more integrative metabolic–thermoregulatory mechanism, whereas Moxotó goats adopt selective and pathway-specific adjustments. Such evidence not only reinforces the adaptive potential of locally adapted breeds but also provides key phenotypic markers to guide conservation and breeding efforts aimed at resilience and sustainability in livestock systems in semiarid ecosystems.

## Data Availability

The datasets generated and analyzed during the current study are available from the corresponding author on reasonable request.

## Code availability

No applicable.
